# Digital divide among higher education faculty

**DOI:** 10.1186/s41239-020-00191-5

**Published:** 2020-04-20

**Authors:** Kamal Ahmed Soomro, Ugur Kale, Reagan Curtis, Mete Akcaoglu, Malayna Bernstein

**Affiliations:** 1 Department of Education, Institute of Business Management (IoBM), Karachi 75190, Pakistan.; 2 39/B, Gulshan-e-Hashmat Bano Town, Phuleli, Hyderabad, Sindh 71000, Pakistan.; 3 Department of Learning Sciences and Human Development, College of Education and Human Services, West Virginia University, Morgantown, WV 26505, USA.; 4 Department of Leadership, Technology & Human Development, College of Education, Georgia Southern University, Statesboro, GA 30458, USA.

**Keywords:** Access to technology, Digital divide, Digital equity, Faculty, ICT

## Abstract

Digital divide centers on access to various dimensions of information and communication technology (ICT) including physical access, motivation, skills, and actual usage of digital technologies. This divide tends to be even wider in the context of developing countries. Yet, there is a lack of literature on the digital divide among the faculty who teach in higher education settings. Thus, as a preliminary effort, by using a 57-item Faculty’s ICT Access (FICTA) scale, we investigated the digital inequalities (at the physical, motivational, skills, and usage levels) among Pakistani faculty in respect of their personal and positional categories. We also examined the relationship between faculty’s instructional usage of ICT and other dimensions of their ICT access. The findings revealed that there were significant differences in the faculty’s access to technology at the four levels in respect of their personal and positional categories. Further, the findings of the study shed light on the theoretical implications of the framework of successive kinds of ICT access suggested by van Dijk (The deepening divide: inequality in the information society, 2005).

Information communications technology (ICT) has the power to raise the quality of people’s lives. It has so much immersed in our life that the digital divide prevents people with no or inadequate ICT access from effective participation in society. The emerging digital technologies embrace the potential of incredible innovation and development prospects (Cruz-Jesus, Vicente, Bacao, & Oliveira, 2016). In recent years, they have postured inducements to increase the involvement of individuals in social, political and economic dimensions of life (Nishijima, Ivanauskas, & Sarti, 2017). ICTs can serve a vital function in the development of all countries (Wardhani, Dugis, & Saad, 2018) and are even more significant for developing countries, for which technology is also being treated as a source to accomplish the United Nations’ Sustainable Development Goals (SDGs) (United Nations, 2015) targets. Nevertheless, to get the most from these technologies, countries should integrate ICT education in their strategic plans (Wardhani et al., 2018).

The digital divide refers to the gap between people who have adequate access to ICT and those who have ‘zero’ or poor access to ICT. Rogers (2016) has referred to this issue as an important issue for social justice in the twenty-first century. Although swift advances in technology have occurred, the digital gap remains ever-present (Centeio, 2017). Such inequalities also exist in educational settings (Centeio, 2017). The existence of the digital gap in different groups related to education such as among teachers and students should be considered as a matter of concern.

The positive and dynamic role of emerging technologies in education has not remained a veiled idea now. Researchers encourage teachers to utilize technology in order to improve their instruction whenever possible (Centeio, 2017). Since technology use in educational settings is considered to be helpful in increasing the access and quality of learning (Domingo & Garganté, 2016), teachers’ access to ICT is of utmost connotation. Digital exclusion will prevent them from taking advantage of technology affordances in their teaching practices. Accordingly, the problem of the digital divide among teachers of all settings needs to be examined. Such investigations help taking necessary measures to remove or at least minimize this problem among educators. They would also help to support the SDGs (United Nations, 2015) which pursue to redress disparity to global access and participation in education.

Studies investigating teachers’ access to digital technologies, explaining specific access types, have not been sufficiently reported in the existing literature, whereas such research in the setting of developing countries just as in Pakistan is totally absent in the literature. Further, the focus of existing work has been mostly limited to measuring physical access to digital devices. However, this issue is more nuanced, involving different facets - motivational, physical, skills and usage access of ICT.

In the present study, we examined Pakistani higher education faculty’s access to ICT regarding their motivational, physical, skills, and usage access. This study particularly focused on the digital gap in terms of the faculty’s access to ICT with respect to their gender, age, and the type of university they teach in. Additionally, the relationship between instructional usage of ICT and various sub-levels of ICT access was explored. The findings are not only supportive in further understanding the impact of demographic variables on digital divide in educational settings but also have theoretical significance to test the digital divide framework suggested by van Dijk (2005).

## Literature review

### Digital Technologies in the Modern era

The present period is referred to as the information age when ICTs are thought to be a prime means of production (Rogers, 2016) and countries’ socioeconomic development is greatly dependent on their access and creation of information. As a consequence of the advancement of information superhighways, our society has undergone rapid and deep changes in social, cultural, political, and economic aspects (Shafique & Mahmood, 2008). ICTs have developed pervasive in society; and they have positively affected every walk of today’s life (Mahmood, 2009), transforming the way people do a job, business, entertain, socialize, and educate.

The ubiquity of ICT in society is considered to act as a booster of socioeconomic development (Hanafizadeh, Hanafizadeh, & Bohlin, 2013; Youssef, Dahmani, & Omrani, 2013). However, access to ICT alone does not assure development in society, but it is people’s reaction that matters once they get access to emerging technologies (Alampay, 2006). As Sianou-Kyrgiou and Tsiplakides (2012) have argued, the socio-economic relationships in the society are structured on the exploitation of information and knowledge rather than on the basis of material goods.

### ICTs in education

Like other aspects of contemporary society such as business, governance, communication, transportation, and entertainment, ICT has evolved into an essential segment of education. In particular, its positive influence on teaching-learning processes is widely acknowledged (Mahmood, 2009). Teachers successfully use technology in their instructional practices for content delivery, reinforcement of students’ skills, complementing the curriculum, and transformation – experimenting, implementing, and refining of new approaches to teaching-learning (Ertmer, Ottenbreit-Leftwich, Sadik, Sendurur, & Sendurur, 2012). Another credible characteristic of emerging technologies is their use for collaboration, which transforms learning into an active and engaging process. The read/write aspect of emerging technologies is especially considered to facilitate students’ learning through sharing knowledge and ideas and practicing collaborative writing (Goh & Kale, 2015).

### Digital divide and its impact on the society

People belonging to different sects of life can contribute their part toward the growth of society in an effective manner if they capitalize on emerging technologies to meaningfully endorse their job and lives. This requires that everyone needs to have physical access to various ICTs and to equip themselves with digital skills. Unfortunately, not all members of society are able to use ICT to participate more effectively for the development of numerous features of the society, due to their uneven access to ICT. This inequality of ICT access generates a multifaceted problem that is known as the digital divide.

Digital divide is a compound and multifaceted problem (Chang, Wong, & Park, 2014). It denotes the divide or gap between the subclasses of the population; the ones that enjoy adequate access to ICT, and the others that have ‘zero’ or poor access to computers, the Internet, and other digital devices. The issue of digital divide prevails, at least to some extent from very large to very small scale. It may contribute to the differences in rich and poor countries, rural and urban areas, men and women, competent and incompetent populations, and micro and macro organizations (Hameed, 2007).

Adequate access to digital technologies can be influential for people to improve their social position and capital. In contrast, the dearth of access to technology can further coerce the already sidelined class of individuals (Rogers, 2016). According to Resta and Laferrière (2015), not only does digital exclusion lead to a knowledge divide but it also confines openings for intercultural networks, communications, and understandings. Considering the negative effects of digital divide on the economically disadvantaged and other marginalized groups, researchers have referred to the problem of the digital gap as a critical issue for social justice in the modern era (Resta & Laferrière, 2015; Rogers, 2016). The issue is present across the globe and continues to be an area of social concern (Resta & Laferrière, 2015).

### The importance of digital equity in education

Initiatives aimed to equip classrooms and build teacher capability in technology use encounter acceptance, sustainability and scalability challenges (Resta & Laferrière, 2015). Among all these issues, the most critical challenge is meeting digital equity among students, teachers, and administrations. For ICTs to empower education, there is a need to launch policies and initiatives that provide students and teachers with equitable access to digital technologies (Resta & Laferrière, 2015). The first and foremost prerequisite for the exploitation of ICT in education is ensuring adequate ICT access by teachers as well as by students. While universities and other higher education institutes are considered as the key sources of skilled workforce upon which a knowledge society is built, the significance of ICT becomes more vivid in universities to help build a knowledge society, making faculty’s ICT access an important area of investigation. Such investigations carry even more significance in emerging countries such as Pakistan, given the higher prevalence of the digital divide problem in their contexts.

**Fig. 1:**
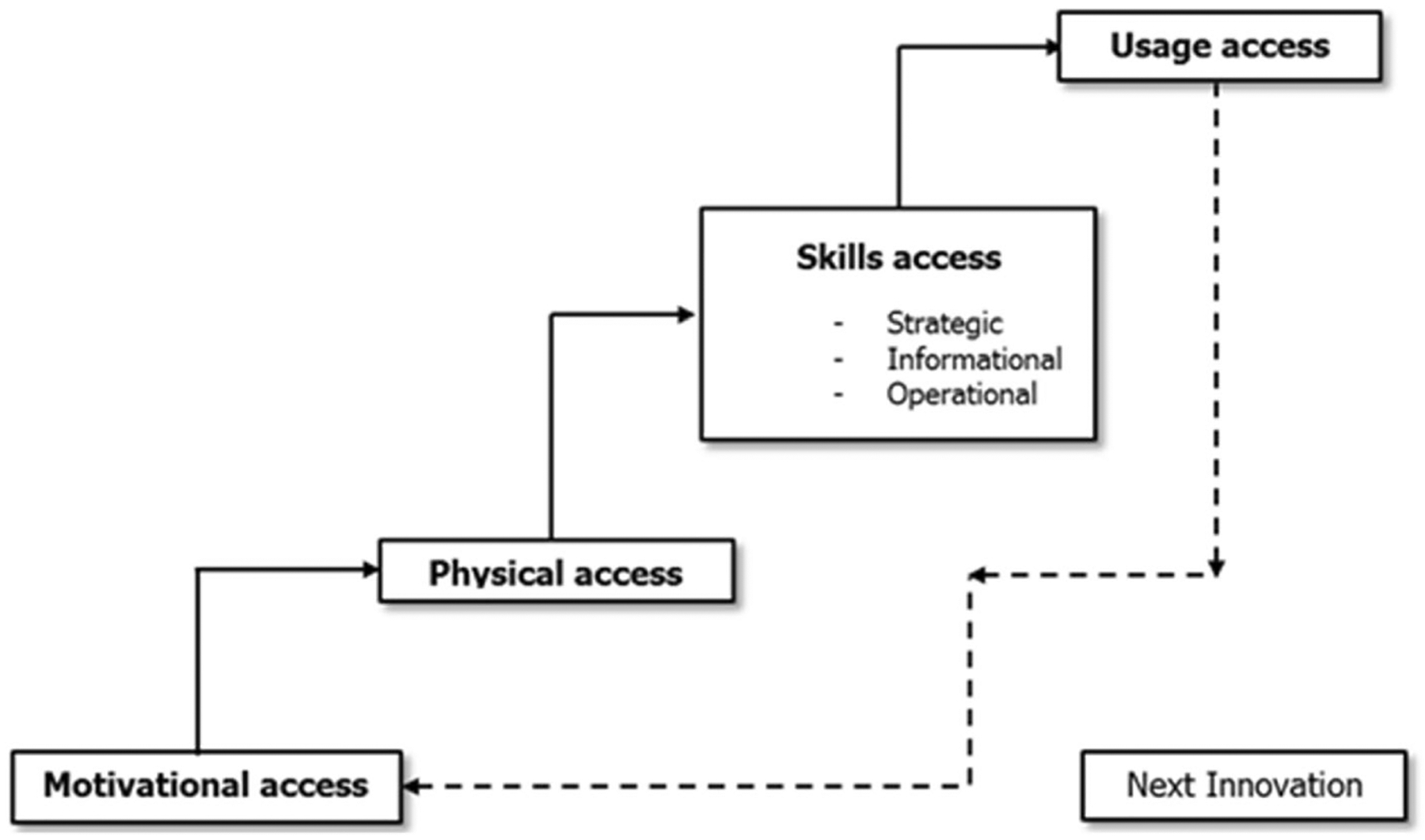
Successive kinds of access to digital technologies (van Dijk, 2005, p. 22)

Apart from the Higher Education Commission of Pakistan’s ICT initiatives to amplify the quality, productivity, and efficiency of academic and research activities in Pakistani universities highlighted in its own reports, there is not sufficient literature available which provides much evidence on technology practices in the universities of the country. As a first step, the present study focuses on examining Pakistani faculty’s ICT access at the four levels (van Dijk, 2005) – their motivations to adopt information and communication technology, their physical access to ICT, their capabilities to utilize digital technologies, and their actual usage of such devices and services. The four levels are the core of van Dijk (2005)’s theory of digital divide, which presented the model of successive kinds of access to ICT (please see Fig. 1), suggesting that there are four successive kinds of access to ICT i.e., motivational, physical, skills, and usage access. The model has classified digital skills into further three types: operational, informational, and strategic skills. The study also provides valuable information on the digital divide among the faculty in respect of their personal and positional categorical variables. Such information would illuminate whether the faculty is in a good position to benefit from the ICT based initiatives taken by HEC Pakistan and to support their teaching and research practices through utilizing emerging technologies.

## Research questions

The present study was based on the following four questions:

1. What are the faculty’s access to digital technologies at four levels (motivational, physical, skills, and usage level)?
2. Are there significant differences among faculty’s access to digital technologies at these four levels?
3. How does faculty’s ICT-access differ with respect to their age, gender, and the type of university?
4. How does faculty’s use of ICT to support their instructional practices relate to their motivational access, physical access, skill access, and general usage access?

## Method

### Research design

To gain insight into the digital divide among higher education faculty, we employed a cross-sectional survey design with a quantitative approach. Data was collected through a self-administered paper-based questionnaire.

### Sampling procedures

The context of the study was the province of Sindh of Pakistan. A purposive sampling approach guided the selection of the universities. We made sure that the selected universities equally represent public and private (non-government) universities. Further, the participating universities included both the general as well as professional universities (medical, engineering, agriculture etc.). After selection of the universities, convenience sampling was used to hire potential participants within the selected universities. The present study employed an anonymous survey – it did not gather any piece of information that might lead to distinctively identify the participants. Participation in the survey was completely voluntary. Moreover, the research method and tool were permitted by the institutional review board of West Virginia University (Protocol# 1412511777).

### Participants

A total of 322 teachers of government and private sector universities teaching in different academic disciplines completed the questionnaire. Table 1 highlights the basic characteristics of the participants.

### Instruments

This study used the Faculty’s Information and Communication Technology Access (FICTA) scale to measure participants’ ICT access (Soomro, Kale, Curtis, Akcaoglu, & Bernstein, 2018). The FICTA scale built on van Dijk’s (2005) model of access to digital technologies (please see Fig. 1). The cronbach alpha confirmed that the scale had acceptable reliability (α = .870).

## Results

This section first presents the findings according to each research question.

### RQ1. Access levels to ICT

#### Motivational access

Participants’ motivational access to ICT was measured by focusing on two different kinds of motivations: endogenous as well as exogenous motivations. Endogenous motivation refers to a person’s desire to adopt ICT that come from *the inside* of the person and is not directly influenced by external sources. Whereas, exogenous motivation focuses on external and contextual aspects, denoting a person’s desire for ICT adoption that come from the outside sources including social influence, time, and material resources. These two constructs were measured through a series of items formatted on a 5-point Likert scale (starting from 1 = strongly disagree to 5 = strongly agree). Scores based on participant’s responses to items in the subscale were averaged to measure each motivation type.

The score for participants’ overall motivation ranged from 1.88 to 4.8, with an average of 3.8 (*SD* = 0.565), which reflects the faculty’s high motivation to adopt digital technologies. The results also showed that Pakistani faculty were motivated to adopt ICT significantly more endogenously (*M* = 4.222, *SD* = 0.537) than exogenously (*M* = 3.38, *SD* = 0.923), *t*(320) = 15.00, *p* < 0.01.

#### Physical access

**Fig. 2:**
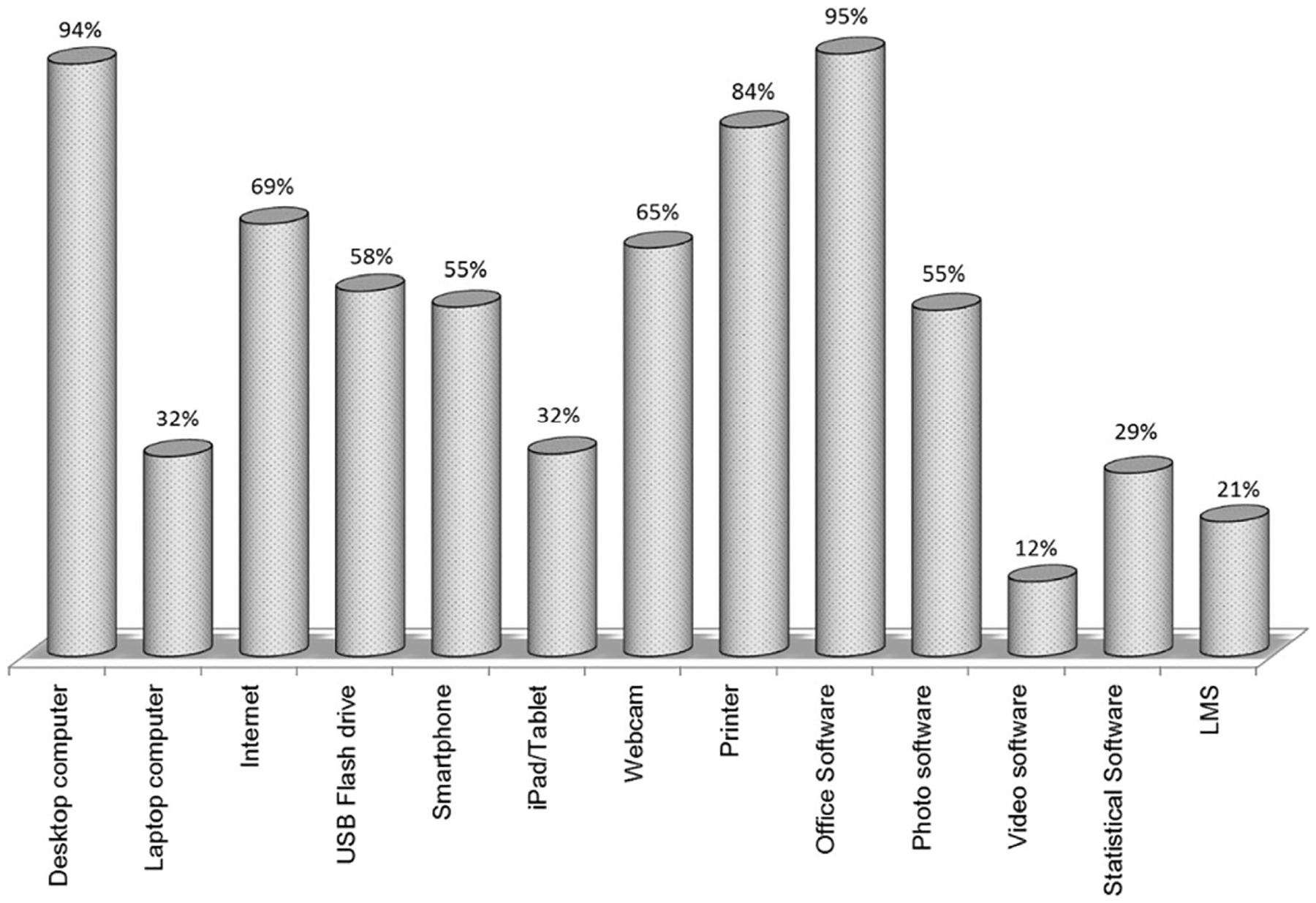
Percentage of participants having physical access to various technologies

The faculty’s physical access to ICT was measured through a checklist comprised of various digital devices, software, etc. Respondents were asked to report whether they had access to the devices given in the list at home and on-campus. Table 2 presents the percentages of faculty who reported to have physical access to various ICT devices and services at home or on-campus. As depicted in Fig. 2, there are a few technologies including a desktop computer, printer, and office software which were accessible by most of the participants. On the other hand, some technologies such as a laptop, tablet, video and statistical software, and learning management system, were accessible by a small proportion of the faculty.

Two separate indexes were computed to reflect participants’ scores for their physical access – access at home and university. Assigning one point to each device, the possible values for a score of physical access at home and university separately ranged from 1 to 13. But, the participant’s score was converted to a scale of 5 points for the purpose of uniformity with other variables. Our results showed that the mean score for the Pakistani faculty’s overall physical access was 2.597 (*SD* = .702). This value within the observed range from .77 to 4.42 indicated faculty’s limited access to a variety of ICT devices and services. Faculty’s physical access was significantly higher at homes (*M* = 2.670, *SD* = .849) than on campus (*M* = 2.528, *SD* = .889), *t*(321) = −2.496, *p* < 0.01.

#### Skills access

Skills access was measured focusing on three types of skills – operational, informational, and strategic skills. The results indicated that participants’ skills level for the three types of skills access differed very slightly, with the mean score of 3.99 (*SD* = .584) for operational skills, 3.904 (*SD* = .549) for informational skills, and 3.8447 (*SD* = .572) for strategic skills.

Further, a composite variable, skills access, was created by computing the average of participant’s score for three kinds of skills, reflecting each participant’s score for his or her overall skills access. The scale ranged from 2.68 to 4.94 and the mean score for Pakistani faculty’s overall skills access was 3.913 (*SD* = .451), indicating their moderate level skills access.

#### Usage access

Usage access was measured focusing on two types of usage: general usage and instructional usage of ICT. The results showed that the faculty’s general usage access (*M* = 3.687, *SD* = .549) was significantly higher than their instructional usage access (*M* = 3.308, *SD* = .616); [*t*(321) = 9.802, *p* < .05]. Further, a composite variable, usage access, was also computed. The scale ranged from 2.19 to 4.53 and the mean score for the faculty’s overall usage access was 3.496 (*SD* = .467), suggesting the faculty had a moderate level of usage access.

The overall score for participants’ access to ICT was calculated by taking the average of their score for each of the four levels described above. The results indicated that the mean score for the faculty’s overall ICT access was 3.448 (*SD* = .316), suggesting that the faculty had a relatively moderate level of ICT access given the scale ranging from 2.77 to 4.21.

### RQ2. Differences in ICT access among the four levels

**Fig. 3:**
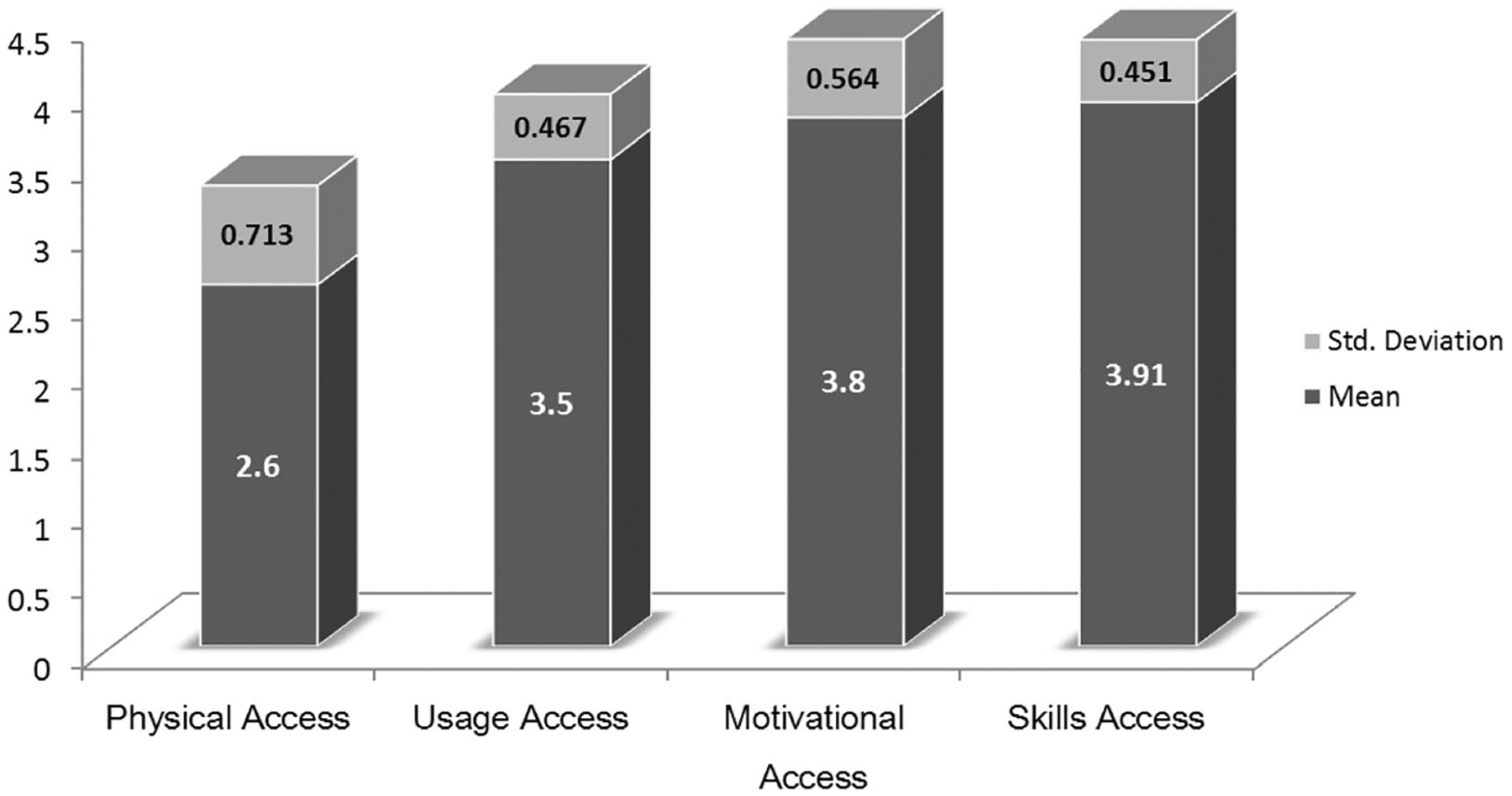
Participants’ access to ICT at the Four Levels

A one-way repeated measure analysis of variance (ANOVA) was conducted to identify potential differences among participants’ ICT access at motivational, physical, skills, and usage levels. The one-way repeated-measures ANOVA compares multiple means when those means have come from the same participants (Field, 2009). The results indicated a significant effect of access levels, Wilks’ Lambda = .230, *F(*3, 315) = 350.706, *p* < .001, eta^2^ = .77. The follow-up comparisons using the Bonferroni correction revealed that each pairwise difference was significant (*p* < .01) except for the pair of motivational and skills access. These results suggest that there were significant differences in participants’ scores for different levels of ICT access. Figure 3 highlights the descriptive statistics for faculty’s access to ICT at the four levels.

### RQ3. Differences in ICT access by age, gender, and university-type

In order to evaluate how well the faculty’s personal and positional categories predict their ICT access, a standard multiple regression was performed (Field, 2009). We performed multiple regressions for each of the four ICT access levels with three predictors: age, gender, and university type (see Table 3). All four regression models were found to be significant (*p* < .001).

Model 1 (motivational access) has the least *R*^2^ value (*R*^2^ = .047, *p* < .001), explaining a small but significant amount of variance in the faculty’s score for the motivational access. The only significant standardized regression weight for gender (*Beta* = −.177, *p* < .001) indicated that gender explained about 5% of the total variance in motivational access (see Table 3). In other words, the model suggested that there were significant differences in the faculty’s motivational access in respect of their gender. The negative sign (−) of the regression weight shows that gender (0 = male and 1 = female) was negatively associated with motivational access, indicating the female faculty members had lower motivational access than their male counterparts.

As Table 3 shows, Model 2 (physical access) explained a significant amount of variance in the faculty’s score for physical access (*R*^2^ = .366, *p* < .001). The model indicated that the university type (*Beta* = .601, *p* < .001) explained about 36% of the total variance in physical access. The model suggested that there were significant differences in the faculty’s physical access with respect to the university type. The faculty working in private sector universities had better physical access to ICT than that of those who teach in public universities. The age and gender were not significant predictors for this model.

Similarly, Model 3 explained a significant amount of variance in the faculty’s score for the skills access (*R*^2^ = .439, *p* < .001). The model indicated that the age (*Beta* = −.291, *p* < .001) and university type (*Beta* = .513, *p* < .001) collectively explained 44% of the total variance in skills access. The model showed that there were significant differences in the faculty’s skills access in respect of the age and university type. In other words, the results suggested that the older the faculty is, the lower their skills access. Similarly, the faculty from private sector universities have higher skills access than their counterparts at public sector universities.

Finally, Model 4 explained a significant amount of variance in faculty’s usage access (*R*^2^ = .276, *p* < .001). The model indicated that the age (*Beta* = −.477, *p* < .001) and gender (*Beta* = −.207, *p* < .001) collectively explained about 28% of the total variance in faculty’s usage access (see Table 3). The model suggested that male faculty’s usage access was higher than the female faculty’s usage access, and younger faculty had significantly higher usage access than that of their older counterpart.

### RQ4. Relationship of instructional usage with other ICT access levels

In order to understand the relationship between the faculty’s instructional use of ICT and their other levels of ICT access, a hierarchical regression analysis was conducted. Table 4 displays two regression models predicting faculty’s instructional usage of ICT. The choice of predictors for these two models was made based on the sequence of the four successive kinds of ICT access (van Dijk, 2005). In the first model, Endogenous Motivational Access and Physical Access at University significantly predicted the faculty’s Instructional Usage of ICT (Adjusted *R*^*2*^ = .196, *F* (4,313) = 20.348, *p* < .001).

In the second model, Operational Skills, Informational Skills, Strategic Skills, and General Usage Access were included in the regression model. Only General Usage Access significantly contributed to the prediction model, with the adjusted *R*^2^ increasing to .221 [*F* (8, 309) = 12.220, *p* < 0.001]. Model 2 resulted in a minor increment (.025) in the adjusted R^2^. The results revealed that the faculty who had a higher score for endogenous motivation, physical access at university, and general usage access were utilizing ICT to support their instructional practices. Out of these three significant predictors, physical access at university (*β* = .338) was the strongest predictor followed by endogenous motivation (*β* = .188) and general usage (*β* = .163) respectively.

## Discussion

In this study, we examined if the faculty’s access to ICT was significantly different at motivational, physical, skills, and usage levels; and to determine if the faculty’s access to ICT significantly differed due to their personal (age, gender) and positional (the type of university) categories. We also explored the relationship of the faculty’s instructional usage of digital technologies with other levels of ICT access.

### A broad view of Pakistani Faculty’s ICT access

The findings of this study provided a broad view of Pakistani faculty’s access to ICT. Our findings suggested that the faculty’s overall ICT access was moderate with an average score of 3.448, while the highest possible score was 5. Despite the emphasis on ICT utilization in educational policies of Pakistan and large investments by higher education commission to initiate various ICT based projects (Ministry of Education, n.d.; Higher Education Commission, 2014; Ministry of Education, 2009), there is still room for improvement in terms of increasing faculty’s ICT access.

Participants were found to have relatively high motivation to adopt and utilize computers, the Internet, and other digital devices and services. An examination of faculty’s mean scores for endogenous motivation and exogenous motivation indicated that they were motivated to adopt digital technologies more because of their own perceptions and attitudes that are internally constructed, rather than being based on external sources such as availability of material resources, time, and social or cultural influence.

Our findings also showed that Pakistani faculty’s physical access to ICT is poor, suggesting they do not have access to adequate ICT infrastructure. Because physical access is an essential condition for the growth of the obligatory skills to practice digital technologies (van Dijk, 2005), the faculty’s inadequate physical access to ICT is alarming. Results also suggested that the faculty had better physical access at home than on their respective campuses. This further calls for attention by concerned authorities to equip university campuses with adequate and latest technologies.

The faculty’s overall skills access to ICT was of moderate level. The means of the three types of skills access were in the order (from highest to lowest) as suggested by the model of successive kinds of technology access (van Dijk, 2005). The faculty’s score for operational skills was highest, followed by informational and strategic skills respectively. Further, the results revealed that the faculty utilizes ICTs mainly to serve general purposes i.e., tasks associated with everyday life other than instructional practices. The faculty’s use of ICT to support their instructional practices such as lesson planning, delivering learning material, facilitating collaboration among students, and evaluating learners’ performance, was relatively low.

### Digital divide regarding personal and positional categories

Our findings showed that there are statistically significant differences in faculty’s access to ICT with respect to their personal and positional categories – age, gender, and type of university. In particular, we found that the overall ICT access of the faculty of public sector universities was lower than that of faculty from private universities. These findings confirm the general perceptions prevailing in the country and are consistent with what Burnip (2006) found in his study with school teachers.

The gap in faculty’s ICT access, between those who work in public-sector and private-sector universities, was prevalent in their physical and skills access, suggesting that the faculty at public sector universities have poorer physical access to ICT devices and services and that they are less competent than their counterparts in the private sector. Lower skills access by the faculty at public sector universities suggests the need for more professional development opportunities in the area of ICT proficiency. With a growing number of universities incorporating technology in teaching-learning, there is a strong need to prepare faculty members for innovative teaching practices (Ranieri, Raffaghelli, & Pezzati, 2018). This could be achieved only when they are not only competent in the general usage of digital technologies but they also know how to use such technologies in teaching their specific subject areas.

The results also confirmed that there is a statistically significant difference in faculty’s access to ICT with respect to their age. The age was significantly negatively associated with skills and usage access to ICT, indicating that younger faculty have higher ICT skills and utilize ICT more often than older faculty do. Thunman and Persson (2013) also found that younger teachers are more inclined to use computers for audio-visual aid in their teaching. Similarly, Soomro, Zai, and Jafri (2015) found that higher education faculty from lower age groups are more competent with Web 2.0 technologies.

Regarding digital divide with respect to gender differences, significant gender differences were observed in faculty’s motivational and usage access. Female faculty were found to be less inclined to adopt digital technologies and had lower usage access than their male counterparts. Improving the use of ICT by females is also emphasized as an important target to achieve the Sustainable Development Goals set by the United Nations (2015) because it helps to promote the empowerment of women (United Nations, 2017). Though previous studies suggested that women are at a disadvantage compared to men in learning computer skills, and more male students use computers at home and university than female students (Cooper, 2006; Mahmood, 2009), our findings did not confirm significant gender differences at physical and skills access to ICT by the faculty. In other words, the results suggested that there is no gap between males and females regarding physical access and skill access. The issue is more about their motivation and usage. These findings partially support van Dijk’s (2005) claims that while gap at lower levels may be closing those at a higher level such as usage may be widening.

### Relationship of instructional usage with other dimensions of ICT access

This study also attempted to explore the relationship between the faculty’s instructional usage of ICT and other dimensions of ICT access. The findings from this study showed that the faculty’s physical access to ICT at university, endogenous motivation, and general usage of ICT significantly predicted instructional usage of ICT. None of the other dimensions of ICT access, including exogenous motivation or any of the three types of skills access, was found to be a significant predictor of faculty’s instructional use of ICT.

These findings suggest that faculty having better ICT infrastructure at their workplace are more inclined to adopt digital technologies to support various dimensions of their instructional practices. Having access to computers and the Internet in their office or campus lab appears to encourage them to utilize technologies to support their primary professional responsibility. Likewise, the positive association of general usage with instructional usage implies that when faculty uses digital technologies for their general tasks other than teaching, they may feel confident and get more ideas on how they should use technology for their teaching. Thus, faculty should be provided with opportunities to learn about general usage ICTs along with the use of technology for instructional purposes. Learning to use digital tools for general purposes is likely to increase their confidence so that it’ll be easier for them to consider them for teaching.

### The relevance of research findings with van Dijk’s theoretical framework (2005)

We found significant differences in the faculty’s ICT access among the four levels. The sequence of faculty’s intensity with the four stages of ICT access (motivational, physical, skills, and usage access) is not in full agreement with what van Dijk (2005) has suggested in his arguments while proposing the model of successive kinds of ICT access. Particularly, higher skills and usage access along with a lower level of physical access does not appear to be fully compatible with the order of succession of the four ICT access levels suggested by the model.

Van Dijk’s (2005) multifaceted model of ICT access suggests that after acquiring sufficient physical access to ICT, one develops his or her capabilities to use digital technologies. This assertion does not imply that once an individual has acquired sufficient physical access to ICT, he or she will develop the digital skills without his or her wish, intention, and efforts to learn digital skills. Further, it does not imply that the intensity with the skills access will be lesser than physical access. Our findings suggest that the faculty’s high motivational access may have helped them to develop better skills to utilize digital technologies, despite their lower physical access to ICT. This supports the affirmation by Ghobadi and Ghobadi (2013) that motivation increases individuals’ skills to utilize ICT.

Ghobadi and Ghobadi (2013) have argued that van Dijk’s (2005) multifaceted model of ICT access is somewhat static, as it does not clarify the interrelations between different levels of ICT access as well as how these levels interact with each other and form digital divide as a whole. According to them, the four access levels are not independent concepts but they are formed as a consequence of complex dynamic interplay with each other. The present study provides a limited understanding of the relationships among the different levels of ICT access. Detailed explanations of the causal processes among the four levels and how each level interacts with others was beyond the scope of this study. To dig deeper into how the four levels of ICT access interact with each other, future research that tests the four types of ICT access simultaneously, by employing path analysis and structural equation modeling may help to better explain how they together create the digital divide.

Additionally, although van Dijk (2006) has affirmed that the digital gap at motivational and physical levels have diminished, and the differences have moved to skills and usage access in the last years, our findings are contradictory to his assertions. The results suggest that the gap exists at all four levels of faculty’s ICT access, including physical and skills access. Even the divide was much bigger in physical access, especially in respect of university type. Therefore, it will be realistic to say that the gap in Pakistani faculty’s physical access to ICT is far from being vanished.

### Limitations and paths for future research

Fully reliance on self-reported data is the primary limitation of this research. The accuracy and validity of the findings are congruent with the participants’ correct understanding of the survey items and their honest responses. Faculty might have been reluctant to report the true picture of their digital skills especially in cases where participants have weak ICT skills. The digital skills of individuals can better be assessed with performance tests. However, the research involving performance tests requires a great deal of time and funds, which makes such investigations difficult to be conducted especially with a large population.

Moreover, we concentrated on the role of personal and positional categories affecting the digital divide among faculty but it did not address other barriers that prevent faculty from adoption and utilization of ICT. Future research can continue to contribute toward a further understanding of the digital gap that arises from those barriers such as lack of professional development opportunities. Last but not least, investigations with a qualitative approach may provide an in-depth understanding on the issue of the digital divide.

## Conclusions

The present study found that ICT access was not universal among the faculty, highlighting the existence of the digital divide with respect to their personal and positional categories including age, gender, and university type. The digital gap between faculty of public-sector and private-sector universities was found to be more prominent at skills access in respect of the faculty’s age and university type; at the physical level in respect of university type; and at usage level in respect of age and gender. Moreover, regarding the relationship between faculty’s instructional usage of ICT and other dimensions of ICT access, the findings suggested that faculty’s physical access to ICT at university, their endogenous motivational access and their general usage access to ICT are the significant predictors of their instructional usage of ICT.

This study is an initial and significant contribution to the literature by portraying a big picture of Pakistani faculty’s motivation to adopt digital technologies. The findings and information gained from this research study provide valuable implications for plans of action for the professional development of faculty and other ICT initiatives in higher education in Pakistan. The findings of the study are also helpful to other researchers in extending understanding of the demographic variables that predict the digital gap among higher education faculty.

## Figures and Tables

**Table 1:** Descriptive statistics for demographic variables

Variable	Coding	Distribution
Age (in years)	Continuous variable	Mean = 38.58
Gender	Male	214 (66.5%)
	Female	108 (33.5%)
Type of university	Public Sector	226 (70%)
	Private Sector	96 (30%)
Teaching experience (in years)	0 to 5	80 (24.8%)
	6 to 10	123 (38.2%)
	11 to 15	75 (23.3%)
	16 to 20	40 (12.4%)
	21 to 25	2 (0.6%)
	More than 25	2 (0.6%)
Teaching position	Lecturer	107 (33.2%)
	Senior Lecturer	34 (10.6%)
	Assistant Professor	120 (37.3%)
	Associate Professor	50 (15.5%)
	Professor	11 (3.4%)

**Table 2:** Summary of the participants’ physical access to ICT devices and services

Technology	Have at home %	Have at university %	Don’t have %
Desktop computer	69.2	73.9	6.2
Laptop computer	26.5	15.4	68.4
Internet	41.4	35.3	31.5
USB flash drive	55.0	14.3	42.1
Smartphone	42.9	37.0	44.6
iPad/Tablet	28.1	7.8	68.0
Webcam	54.3	17.8	35.3
Printer	44.4	77.3	16.2
Office software	77.0	79.5	4.6
Photo editing software	48.8	39.9	45.1
Video editing software	22.4	16.8	88.2
Statistical software	25.5	43.7	71.0
Learning management system	14.9	17.4	78.7

**Table 3:** Summary of regression results predicting the four levels of ICT access

Regression Models	*R* ^2^	Predictors		
		Age	Gender	University type
Model 1 (Motivational access)	.047*	−.096	−.177**	.086
Model 2 (Physical access)	.366*	−.027	−.040	.601*
Model 3 (Skill access)	.439*	−.291*	−.059	.513*
Model 4 (Usage access)	.276*	−.477*	−.207*	.031

* *p* < .001,

** *p* < .01

**Table 4:** Hierarchical regression results predicting instructional usage access (N = 317)

Variables	*Model 1*				*Model 2*			
	*B*	*SE*	*β*	*t*	*B*	*SE*	*β*	*t*
(Constant)	1.874	.306		6.129***	1.344	.418		3.212**
Endogenous motivational	.206	.060	.180	3.453**	.215	.059	.188	3.621***
Exogenous motivational	.047	.034	.070	1.361	.038	.034	.057	1.110
Physical access at university	.285	.038	.413	7.596***	.233	.042	.338	5.542***
Physical access at home	.001	.040	.001	.026	.012	.040	.016	5.542
Operational skills					.067	.065	.063	1.018
Informational skills					.066	.063	.059	1.052
Strategic skills					.109	.072	.102	1.507
General usage					.182	.059	.163	3.070**
*R*	.454				.490			
*R* ^*2*^	.206				.240			
*Adjusted R* ^*2*^	.196				.221			
*Adjusted R*^*2*^ *change*	.196				.025			
*F*	20.348***				12.220***			

* *p* < .05,

** *p* < .01,

*** *p* < .001

## Data Availability

All data of this research project is available with the authors and can be made available on requests if required.

## Appendix

### 57-item FICTA scale

**Table:**

Physical access
1. Desktop computer2. Laptop computer3. Broadband/DSL internet4. USB Flash drive (memory stick)5. Smartphone (cell phone internet functionality)6. iPad/Tablet7. Webcam8. Printer9. Office Software Suit (e.g., Microsoft Office, Open Office)10. Photo editing software (e.g. Adobe Photoshop, Corel Paint)11. Video editing software (e.g. iMovie, Movie Maker)12. Statistical Software (e.g., SPSS, SAS)13. Learning Management System (e.g., Blackboard, eCampus)
Endogenous motivational access
14. Using the Internet can provide me with information that would lead to better decisions.15. Using ICT will be of no benefit to me.16. Using the computer and the Internet can improve my work performance.17. Using a Computer and the Internet seems to be enjoyable.
Exogenous motivational access
18. Seeing other teachers using computers and the Internet inspires me.19. I want to use ICT because my superiors expect me to use it.20. I wish to use a computer and the Internet because my students think that I should use them.21. I am interested in adopting digital technologies because my university provides enough technology support.
Operational skills access
22. I feel comfortable in creating and editing a text file in a word processing program.23. It is easy for me to create a computer presentation.24. I feel difficulty to change some basic computer settings (wallpaper, time/date, sounds, etc.).25. I can save images and text from the website on the hard disk.26. I feel confident to download programs from the internet.27. I can send an attachment to an email.28. I know enough about transferring files from hard disk to a USB flash drive and vice versa.
Informational skills access
29. I always know what search terms to use when searching the internet.30. I can use advance search options to reach my required information.31. I feel confident to evaluate the sources of the information found on the Internet.32. I feel comfortable synthesizing online information.33. It is easy for me to retrieve a Website on the Internet.34. I can easily choose from search results.
Strategic skills access
35. I can make a choice by consulting the Internet.36. I can reach my intended goal while using the Internet.37. On the Internet, it is easy for me to work toward a specific goal.38. I can gain benefits from using the computer and the Internet.39. Using various ICT tools, I feel confident in achieving my goals.40. I feel confident in making important decisions with the help of the Internet.
General usage access
41. I search for the information of my interest on the Internet.42. I use ICT to support my research activities.43. I use emails as one of the primary means of communication.44. I make voice/video calls via the Internet.45. I create letters, reports and/or papers on computer.46. I prepare presentations on computer.47. I store and manipulate data in a spreadsheet program.48. I use digital technologies to watch movies or television programs.
Instructional usage access
49. I use ICT for communication about assignments among students.50. I use ICT for enhancing students’ content learning.51. I use ICT for facilitating students’ group work.52. I use ICT to improve students’ problemsolving skills.53. I use digital technologies for the delivery of my instruction.54. I use digital technologies to communicate with students.55. I prepare learning materials using computer and internet resources.56. I develop critical thinking skills among students with the help of ICT.57. I use ICT to encourage peer-feedback among my students.

## References

[R1] Alampay E (2006). Beyond access to ICTs: measuring capabilities in the information society. International Journal of Education and Development Using ICT, 2(3), 4–22.

[R2] Burnip L (2006). ICT-mediated study and teachers: do they have access to the infrastructure? Australasian Journal of Educational Technology, 22(3), 355–374.

[R3] Centeio EE (2017). The have and have nots: an ever-present digital divide. Journal of Physical Education, Recreation & Dance, 88(6), 11–12. 10.1080/07303084.2017.1331643.

[R4] Chang Y, Wong SF, & Park MC (2014). A three-tier ICT access model for intention to participate online a comparison of developed and developing countries. Information Development. 10.1177/0266666914529294.

[R5] Cooper J (2006). The digital divide: the special case of gender. Journal of Computer Assisted Learning, 22(5), 320–334.

[R6] Cruz-Jesus F, Vicente MR, Bacao F, & Oliveira T (2016). The education-related digital divide: an analysis for the EU-28. Computers in Human Behavior, 56, 72–82. 10.1016/j.chb.2015.11.027.

[R7] Domingo MG, & Garganté AB (2016). Exploring the use of educational technology in primary education: teachers’ perception of mobile technology learning impacts and applications’ use in the classroom. Computers in Human Behavior, 56, 21–28. 10.1016/j.chb.2015.11.023.

[R8] Ertmer PA, Ottenbreit-Leftwich AT, Sadik O, Sendurur E, & Sendurur P (2012). Teacher beliefs and technology integration practices: a critical relationship. Computers & Education, 59(2), 423–435. 10.1016/j.compedu.2012.02.001.

[R9] Field A (2009). Discovering statistics using SPSS. London: Sage publications.

[R10] Ghobadi S, & Ghobadi Z (2013). Digital divide and interrelated access gaps: a cognitive investigation. Utrecht: Paper presented in European Conference on Information Systems Retrieved from http://www.staff.science.uu.nl/~vlaan107/ecis/files/ECIS2013-0338-paper.pdf.

[R11] Goh D, & Kale U (2015). The urban–rural gap: project-based learning with web 2.0 among West Virginian teachers. Technology, Pedagogy and Education, 25, 1–22. 10.1080/1475939X.2015.1051490.

[R12] Hameed T (2007). ICT as an enabler of socio-economic development. Seoul: Paper presented at the Digital Opportunity Forum Retrieved from http://unpan1.un.org/intradoc/groups/public/documents/un-dpadm/unpan043799.pdf.

[R13] Hanafizadeh MR, Hanafizadeh P, & Bohlin E (2013). Digital divide and e-readiness: trends and gaps. International Journal of E-Adoption (IJEA), 5(3), 30–75.

[R14] Higher Education Commission (2014). Annual report 2012–13. Retrieved December 16, 2016, from http://hec.gov.pk/MediaPublication/Documents/Annual%20Reports/Annual%20Report%202012-13.pdf

[R15] Mahmood K (2009). Gender, subject and degree differences in university students’ access, use and attitudes toward information and communication technology (ICT). International Journal of Education and Development using Information and Communication Technology, 5(3), 206–216.

[R16] Ministry of Education (2009). National education policy 2009. Government of Pakistan: Ministry of Education.

[R17] Ministry of Education (n.d.). National information and communications technology strategy for education in Pakistan. Government of Pakistan: Ministry of Education.

[R18] Nishijima M, Ivanauskas TM, & Sarti FM (2017). Evolution and determinants of digital divide in Brazil (2005–2013). Telecommunications Policy, 41(1), 12–24. 10.1016/j.telpol.2016.10.004.

[R19] Ranieri M, Raffaghelli J, & Pezzati F (2018). Digital resources for faculty development in e-learning: a self-paced approach for professional learning. Italian Journal of Educational Technology, 26(1), 104–118.

[R20] Resta P, & Laferrière T (2015). Digital equity and intercultural education. Education and Information Technologies, 20(4), 743–756. 10.1007/s10639-015-9419-z.

[R21] Rogers SE (2016). Bridging the 21st century digital divide. TechTrends, 60(3), 197–199. 10.1007/s11528-016-0057-0.

[R22] Shafique F, & Mahmood K (2008). Indicators of the emerging information society in Pakistan. Information Development, 24(1), 66–78.

[R23] Sianou-Kyrgiou E, & Tsiplakides I (2012). Digital divide: students’ use of the internet and emerging forms of social inequalities. In Jimoyiannis A (Ed.), Research on e-learning and ICT in education, (pp. 55–68). New York: Springer.

[R24] Soomro KA, Kale U, Curtis R, Akcaoglu M, & Bernstein M (2018). Development of an instrument to measure faculty’s information and communication technology access (FICTA). Education and Information Technologies, 23(1), 253–269. 10.1007/s10639-017-9599-9. 29375248 PMC5784782

[R25] Soomro KA, Zai SY, & Jafri IH (2015). Competence and usage of web 2.0 technologies by higher education faculty. Educational Media International, 52(4), 284–295. 10.1080/09523987.2015.1095522.

[R26] Thunman E, & Persson M (2013). Teachers’ access to and use of ICT: an indicator of growing inequity in Swedish schools. Contemporary Educational Technology, 4(3), 155–171.

[R27] United Nations (2015). Sustainable development goals. Retrieved March 12, 2019 from: https://sustainabledevelopment.un.org

[R28] United Nations (2017). Sustainable development goals indicators. Retrieved March 12, 2019 from: https://unstats.un.org/sdgs/indicators/indicators-list/

[R29] van Dijk JAGM (2006). Digital divide research, achievements and shortcomings. Poetics, 34(4), 221–235.

[R30] van Dijk JAJGM (2005). The deepening divide: inequality in the information society. Thousand Oaks: Sage Publications.

[R31] Wardhani B, Dugis V, & Saad MS (2018). On the digital divide: role of the University of the South Pacific in enhancing education in the Pacific countries. World Transactions on Engineering and Technology Education, 16(1), 36–41.

[R32] Youssef AB, Dahmani M, & Omrani N (2013). Information technologies, students’ e-skills and diversity of learning process. Education and Information Technologies, 20(1), 141–159. 10.1007/s10639-013-9272-x.

